# A scoping review of patient safety research carried out in the Republic of Ireland

**DOI:** 10.1007/s11845-022-02930-1

**Published:** 2022-02-05

**Authors:** Paul O’Connor, Roisin O’Malley, Yazeed Kaud, Emily St. Pierre, Rosie Dunne, Dara Byrne, Sinéad Lydon

**Affiliations:** 1 Department of General Practice, School of Medicine, National University of Ireland Galway, 1 Distillery Road, Galway, Co Ireland; 2 Irish Centre for Applied Patient Safety and Simulation, National University of Ireland Galway, Galway, Ireland; 3 School of Medicine, National University of Ireland Galway, Galway, Ireland; 4 Department of Public Health, Saudi Electronic University, Riyadh, Saudi Arabia; 5 James Hardiman Library, National University of Ireland Galway, Galway, Ireland

**Keywords:** Ireland, Patient safety, Research, Scoping review

## Abstract

Maintaining the highest levels of patient safety is a priority of healthcare organisations. However, although considerable resources are invested in improving safety, patients still suffer avoidable harm. The aims of this study are: (1) to examine the extent, range, and nature of patient safety research activities carried out in the Republic of Ireland (RoI); (2) make recommendations for future research; and (3) consider how these recommendations align with the Health Service Executive’s (HSE) patient safety strategy. A five-stage scoping review methodology was used to synthesise the published research literature on patient safety carried out in the RoI: (1) identify the research question; (2) identify relevant studies; (3) study selection; (4) chart the data; and (5) collate, summarise, and report the results. Electronic searches were conducted across five electronic databases. A total of 31 papers met the inclusion criteria. Of the 24 papers concerned with measuring and monitoring safety, 12 (50%) assessed past harm, 4 (16.7%) the reliability of safety systems, 4 (16.7%) sensitivity to operations, 9 (37.5%) anticipation and preparedness, and 2 (8.3%) integration and learning. Of the six intervention papers, three (50%) were concerned with education and training, two (33.3%) with simplification and standardisation, and one (16.7%) with checklists. One paper was concerned with identifying potential safety interventions. There is a modest, but growing, body of patient safety research conducted in the RoI. It is hoped that this review will provide direction to researchers, healthcare practitioners, and health service managers, in how to build upon existing research in order to improve patient safety.

## Introduction

A commitment to improving safe healthcare features in governmental policies worldwide. However, progress in delivering on this aspiration has been modest, with patients still suffering avoidable harm [1]. A major challenge to improving safety is the lack of high-quality information to allow healthcare organisations, teams, and individuals to evaluate how they are performing, and where there are deficits and risks [2]. This safety information is complex and multi-faceted, yet vitally important if safety is to improve [3].

In the Republic of Ireland (RoI), “maintaining the highest levels of patient safety is a fundamental priority for patients and for healthcare organisations”(p.5) [4]. The need for proactive approaches to patient safety has been identified by the Irish Health Service Executive (HSE) [4]. There is a recognition that such an approach requires high-quality data that will support learning from patient safety incidents, identification of hazards or risks, and the implementation of interventions to improve safety [4]. It is only through effective measurement and monitoring of safety (MMS) that comparisons can be made between the safety performance of different healthcare organisations, the impact of safety interventions can be assessed, and there can be a shift to a more proactive approach to safety.

In addition to efforts to improve the MMS, there is also a need to consider the effectiveness of patient safety interventions. There has been considerable investment in patient safety improvement efforts, for which there may be limited evidence of effectiveness [5]. It has been found that the majority of safety interventions tend to be person-focused (e.g. education and training), with more effective systems focused interventions far less commonplace [6]. Moreover, high-quality research on the effectiveness of safety intervention is lacking [5]. Therefore, there is a need for rigorous assessment of the effectiveness of interventions to ensure that they are having the desired effect, and the resources required to implement such interventions are justified. Crucially, given the recognised impact of context on intervention implementation and effectiveness, such assessments must be conducted within different healthcare systems and services [7].

The purpose of this scoping review is to examine the extent, range, and nature of patient research activities carried out in the RoI. Research is fundamental to improving practice, particularly within an applied science such as patient safety [8]. Accordingly, the findings from this review will be used to make recommendations for future patient safety research, and the alignment between these recommendations and the HSE patient safety strategy 2019–2024 [4] will be delineated.

## Methods

This scoping review is conducted using the five-stage approach proposed by Arksey and O’Malley [9] and reported according to the Preferred Reporting Items for Systematic Reviews and Meta-Analyses extension for Scoping Reviews (PRISMA-ScR) checklist [10]. Scoping reviews provide an increasingly popular option for synthesising and mapping evidence in healthcare research [11].

### Stage 1: Identify the research question

The purpose of the review was clearly defined with concept of interest (i.e. patient safety research), target population (i.e. healthcare staff and patients in secondary care), and location (i.e. RoI).

### Stage 2: Identify relevant studies

#### Search strategy

Electronic searches were conducted across five electronic databases in July 2021: Medline, CINAHL, Embase, PsycInfo, and Web of Science. The search strategy was finalised by a Research Librarian (RD). The search strategy comprised Medical Subject Headings terms along with free-text keywords, and was altered as necessary for the remaining databases (see Supplementary Data 1 [12] for the Medline search strategy). In addition to electronic searches, the reference lists of all studies identified as eligible for inclusion from the electronic searches were screened to identify any other potentially suitable articles.

### Stage 3: Study selection

Titles and abstracts of all articles identified during the electronic searches were screened by one of three authors (ROM, YK, or ESP) in July 2021. The full-texts of articles that appeared eligible for inclusion, or articles in which the title and abstract did not provide sufficient information for the determination to be made, were reviewed in full to confirm their eligibility. For papers where inclusion was unclear, all members of the research team reviewed the paper, and decisions on eligibility were made through discussion.

#### Inclusion criteria

Inclusion criteria required that studies: (1) were focused on patient safety in hospitals in the RoI including, but not limited to, the measurement of safety or implementation of initiatives aimed at improving safety; (2) reported original research; (3) were published in a peer-reviewed journal; and (4) were written in English.

#### Exclusion criteria

Studies were excluded if they: (1) focused on patient safety in the context of patients with a particular medical condition only (e.g. patients with cancer); (2) focused on the safety of one process only (e.g. medication errors); (3) were conducted in healthcare settings other than hospitals; (4) were conducted in a country other than the RoI or a sample of countries including the RoI where RoI-specific data could not be extracted; (5) only employed one item/question relating to patient safety as part of a larger survey or assessment (i.e. studies had to use a full measure of patient safety); or (6) did not report original research. No limits were placed on the publication year.

### Stage 4: Chart the data

A preliminary data charting form was developed in accordance with best practice [13], and piloted by two authors (YK, ROM). The form was used to extract data on author(s), year of publication, study location, study aim, methods, sample, intervention (if included), comparator (if included), outcome measures, and key reported outcomes. Data were extracted by three authors (ROM, YK, and ESP), with two of these authors extracting data independently for each included article. Disagreements were resolved through discussion.

### Stage 5: Collate, summarise, and report the results

The characteristics of the included studies were collated and summarised across several key descriptors: location; aim; methods; sample; type and duration of intervention (if applicable); comparators (if applicable); outcome measures; and key outcomes.

Included studies were summarised according to one of two different frameworks. Studies that involved MMS were categorised using the five domains of Vincent et al. [3, 14] MMS framework (see Table 1). It was possible for both studies and measures described to be categorised under more than one MMS dimension.

**Table 1:** Description of the MMS and hierarchy of intervention effectiveness frameworks

**MMS framework** [3, 14]
1. *Harm*: has patient care been safe in the past? (e.g. case record review, patient safety indicators) [14] 2. *Reliability of safety critical processes*: are our clinical systems and processes reliable? (e.g. audit of equipment availability, observations of safety critical behaviour) [14] 3. *Sensitivity to operations*: is care safe today? (e.g. talking to patients, ward rounds) [14] 4. *Anticipation and preparedness*: will care be safe in the future? (e.g. human reliability analysis, safety culture assessment) [14] 5. *Integration and learning*: are we responding and improving? (e.g. regular integration and review by clinical teams, feedback and implementation of safety lessons) [14]
**Hierarchy of intervention effectiveness framework** [15]
1*. Forcing functions*: designing processes so that errors are virtually impossible or difficult to make (e.g. removing potassium chloride for injection concentrate from all patient care areas) [15] 2. *Automation and computerisation*: automating and computerising processes and tasks to lessen human fallibility by limiting reliance on memory (e.g. use of technologically, computerised drug information systems) [15] 3. *Simplification and standardisation*: developing and implementing protocols and standardised order forms to guide the safety of processes by eliminating problems with illegible handwriting and standardising safe order communication (e.g. development of protocol for transferring patients) [15] 4. *Reminders, checklists, and double checks*: developing tools that can reduce the risk of error by standardising processes and/or having one person independently check another’s work (e.g. independent double check systems) [15] 5. *Rules and policies*: establishing and enforcing rules and policies related to error prevention and safety (e.g. implementing policies around safe medication use) [15] 6.*Education and training*: educating and training healthcare staff to reduce errors and to promote and ensure patient safety (e.g. intervention on improving attitudes towards patient safety) [15]

Studies of a safety intervention were classified using the hierarchy of intervention effectiveness framework [15] (see Table 1). The framework delineates interventions according to six levels of effectiveness from 1 (most effective) to 6 (least effective). The hierarchy of intervention effectiveness framework was first discussed by the Institute for Safe Medication Practices, and has since been referenced a number of patient safety organisations as an approach to guide the identification of suitable safety interventions (e.g. Incident Analysis Collaborating Parties [16], Health Information and Quality Authority [17]). The hierarchy of interventions was extended by Woods et al. [18], who added three additional levels (staff organisation, risk assessment, learning from errors, and personal initiative) as this was deemed necessary in order to appropriately classify solutions to improving clinical communication and patient safety. However, for the purposes of this scoping review, we used the original six level framework due to our focus on interventions, rather than solutions (see Table 1).

The categorisation of study content via these two frameworks was carried out independently by three reviewers (ROM, YK, and ESP). Where disagreements arose, the study was discussed by all members of the review team and a decision on the categorisation was made by consensus. Following completion of all data charting and coding, the meaning of the findings and their implications were appraised within the context of the broader literature in this area, and the HSE patient safety strategy [4].

## Results

**Fig. 1:**
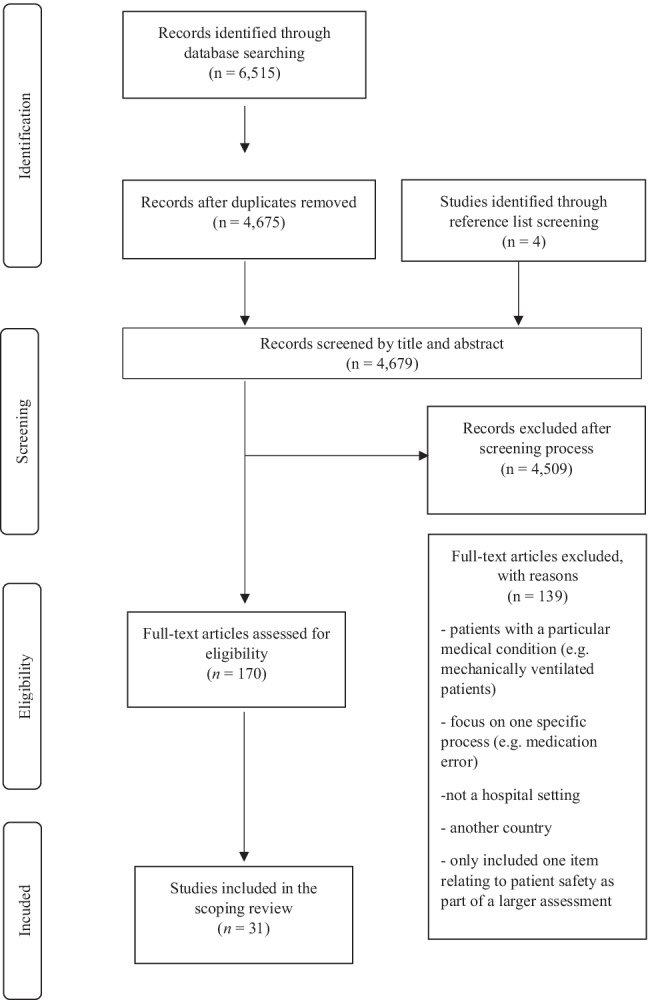
PRISMA flowchart of the search and screening process

A total of 6515 articles were identified from electronic database searches (see Fig. 1), with 170 full-texts examined and 27 papers ultimately meeting the inclusion criteria. Four additional studies were identified through reference list screening, resulting in the inclusion of 31 studies (published 2003–2021). Study characteristics are outlined in Table 2, and a summary of the main findings from the studies is provided in Table 3.

**Table 2:** Summary of the characteristics of the included studies

C**haracteristics**	**Studies *n* (%)**
**Year of publication**	
2000–2004	1 (3.2)
2005–2008	0 (0)
2009–2012	7 (22.6)
2013–2016	8 (25.8)
2017–2020	12 (38.7)
From January to May 2021	3 (9.7)
**Type of data collected**	
Quantitative	23 (74.2)
Qualitative	4 (12.9)
Quantitative and qualitative	4 (12.9)
**Categorisation of MMS studies (*n* = 24)**	
Past harm	12 (38.7)*
Reliability of safety critical processes	4 (12.9)
Sensitivity to operations	4 (12.9)
Anticipation and preparedness	9 (29.0)
Integration and learning	2 (6.5)
**Categorisation of intervention studies (*n* = 6)**	
Forcing functions	0 (0)
Automation and computerisation	0 (0)
Simplification and standardisation	2 (6.5)
Reminders, checklists, and double checks	1 (3.2)
Rules and policies	0 (0)
Education and training	3 (9.7)
**Other types of study (*n* = 1)**	
Intervention development study	1 (3.2)

^*^These figures do not total to 24 because some of the studies related to more than one dimension of the MMS framework (3, 13)

**Table 3:** Summary of key findings resulting from included MMS and intervention studies

Categories	Key findings
Past harm	• Adverse events are not uncommon [1, 19, 20, 23–25] • The prevalence of adverse events was 12.2% in 2009 [1] and 14% in 2015 [25] • The prevalence of preventable adverse events was 9.1% in 2009 [1] and 7.4% in 2015 [25] • Slips/trips and falls account for the majority (32%) of all adverse outcomes reported with medication errors and perioperative incidents making up the 2nd and 3rd most common adverse events respectively [29] • The economic cost of adverse events to the health service in Ireland is estimated to be between €91.3 [26] and €194 million [25] • Ireland had greater than the mean number of secondary diagnoses for three out of five patient safety indicators: catheter-related bloodstream infection; postoperative pulmonary embolism (PE) or deep vein thrombosis (DVT); and postoperative sepsis rates. Ireland was below the mean for accidental puncture or laceration, and foreign body left in during procedure [27] • Across surgical specialties, the majority of reported adverse events occur in orthopaedic and general surgery (73% of all claims) [29] • Nurses and midwives report adverse events with a much greater frequency than doctors [29] • Reluctance to report adverse events is influenced by fears of retribution [23, 24] • A survey of junior doctors found that 60.5% of respondents reported making an error that “played on their mind” [28] • Burnout is associated with higher rates of self-reported medical error [21, 22]
Reliability of safety critical processes	• The use of surgical checklists was high in Ireland [30, 32] • Participating in Time Out as a team was reported as occurring in 57% of cases [30] • Although attitudes towards the effect of the checklist on safety and teamwork were positive [30, 31], barriers to use such as lack of time were reported [30, 31]—particularly among nurses [31]
Sensitivity to operations	• Healthcare providers described the poor working conditions in the hospital, but also recognised the importance of teamwork and communication in maintaining patient safety and had a strong appetite for change regarding the safety culture in the hospital [34] • 8–9% nurses gave their hospital a poor or failing safety grade [19, 20, 33]
Anticipation and preparedness	• Studies that used the Safety Attitudes Questionnaire (SAQ) found that hospitals scored higher than international benchmarks in the domains: “Teamwork Climate”[35–37]; “Safety Climate”[35–37]; “Job Satisfaction”[35–37], “Stress Recognition”[35–37]; “Perceptions of Management”[35–37]; and “Working Conditions”[35, 37] • At ward level, factors such as the ward practice environment and the proportion of nurses with degrees were found to significantly impact safety outcomes [20] • Nurses’ main concern was how to minimise risk [38, 39] • Many healthcare providers reported not feeling supported by hospital management [34], and doubted that safety was a management priority [19] • In situ simulation was used to identify latent safety hazards [40] • Over 85% of staff liked their job and would feel safe being treated at the hospital as a patient [35]
Integration and learning	• Statistically significant changes in clinical activity were identified in the 28 days following five of the six severe perinatal adverse events [41] • A steady improvement in transfer time was demonstrated between the first and last simulation of a series of four simulations aimed at identifying latent safety hazards [40]
Intervention studies	• The percentage adherence to the Good Surgical Practice Guidelines was higher in an intervention group that received an adhesive ward round checklist (91%) in comparison with the control group (55%)[47] • Participating in the Online Patient Safety Education Programme resulted in immediate improvement in skills such as knowing when and how to complete incident forms and disclosing errors to patients, in self-rated knowledge and attitudes towards error reporting [44] •Of 72 incident forms received in the first 4 months of the Clinical Risk Management project, 25.3% related to actual clinical incidents and 12.6% related to near misses. Potential risk was present in 62% of the reports [45] • The implementation of a 30-day complication proforma led to a 73% increase in morbidities reported using the proforma as compared with traditional Morbidity and Mortality reporting (547 vs 316), and an increase of 10.8% in the reporting of mortalities [46] • The implementation of training based on Crew Resource Management was associated with a significant increase in knowledge as a result of the training, and there was some evidence to support a shift in attitudes in the desirable direction relating to the need to speak up to seniors. No effect of the training was found on behaviour [43] • A significant change in the reporting behaviour of junior doctors was observed in one of the two hospitals following the intervention, a serious board game “PlayDecide patient safety” [42]

### Studies focused on past harm

Past harm was the most frequently assessed dimension of the MMS framework, and was measured in 12 studies (see Tables 2 and 3, and Online Supplementary Material 2 [12]). Six studies employed surveys to measure past harm. Two of these studies used surveys to estimate the frequency of a range of adverse events [19] and to examine nurse adverse event reporting rates [20]. Of the four remaining studies that used a survey design, two examined the association of burnout with self-reported medical error and poor-quality care [21, 22], and two studies explored nurse incident reporting [23, 24]. Four studies measured past harm by retrospectively reviewing patient records. Two of these record reviews were undertaken as part of the Irish National Adverse Events studies [1, 25], and examined trends in adverse event rates in the Irish healthcare system. The two remaining record reviews were conducted to estimate the economic cost of nurse-sensitive adverse events [26] and to compare the health system performance of 15 Organisation for Economic Co-operation (OECD) countries across seven patient safety indicators [27]. Furthermore, one study used a combination of survey and interview methods to examine the nature and frequency of medical error among junior doctors [28], and one study comprised a review of medico-legal claims to identify current adverse event reporting trends in Irish surgical specialties [29].

### Studies focused on reliability of safety critical processes

Four studies assessed the reliability of safety critical processes (see Tables 2 and 3, and Online Supplementary Material 2 [12]). Of the two studies that used a survey design to monitor reliability, one study employed surveys to examine the implementation of Surgical Safety Checklists (SSC) in Irish operating theatres [30] while the other study used interviews to develop a survey evaluating the attitudes of theatre staff towards a surgical checklist [31]. Two studies used patient record review methodology to assess reliability, one of which reviewed patient records to assess the prevalence of surgical checklist use in Europe [32] while the other study used hospital data to improve the international comparability of patient safety indicators [27].

### Studies focused on sensitivity to operations

Four studies included a measure that assessed sensitivity to operations (see Tables 2 and 3, and Online Supplementary Material 2 [12]). Three of these studies used surveys and asked nurses to give their ward an overall safety grade [19, 20, 33]. One study conducted interviews to explore aspects of safety culture that were important to the staff at the time of the interviews [34].

### Studies focused on anticipation and preparedness

Almost a third of the included studies focused on anticipation and preparedness (see Tables 2 and 3, and Online Supplementary Material 2 [12]). Five studies used surveys to assess patient safety culture. Three of these studies employed the Safety Attitudes Questionnaire (SAQ) [35–37], and two studies used items from other surveys [19, 20]. Interviews and/or observations were used by three studies to investigate healthcare workers’ perceptions of the safety culture [34] and to explore how nurses promote safety in perioperative settings [38, 39]. One study used in situ simulation to examine latent safety hazards in response to preparation for an expected COVID-19 surge [40].

### Studies focused on integration and learning

Integration and learning was assessed by two studies (see Tables 2 and 3, and Online Supplementary Material 2 [12]). McNamara and O' Donoghue [41] reviewed patient records to objectively demonstrate if a change in labour ward clinical activity occurred following serious adverse perinatal events. Jee et al. [40] identified system errors and latent safety hazards using in situ simulation and described the resulting corrective measures taken to improve their pandemic response locally.

### Intervention studies

Six studies were categorised as intervention studies. Studies employed several different types of intervention of varying effectiveness (see Tables 2 and 3, and Online Supplementary Material 2 [12]). Three studies comprised interventions that focused on improving patient safety through education and training [42–44]. One of these studies implemented a board game to educate junior doctors about patient safety and the importance of reporting safety concerns [42]. The second educational intervention was concerned with training aimed at improving interns’ attitudes towards, and ability to, “speak up” to senior physicians [43], and the third comprised an online patient safety education programme for junior doctors [44].

Two of the studies implemented interventions focused on improving safety through simplification and standardisation. Both of these studies involved the implementation of an incident/near miss reporting form [45] or complication proforma [46]. Finally, one study sought to improve patient safety by implementing an intervention focused on reminders, checklists, and double checks. This intervention involved the development and implementation of an adhesive surgical ward round checklist [47].

There was one study included in the review that was not concerned with MMS or constituted an intervention itself. Rather, the focus of this study was on the development of a collective leadership intervention for healthcare teams to improve team performance and patient safety culture [48].

## Discussion

This scoping review has demonstrated that, although overall modest in size, there is a growing body of research on patient safety in the RoI published in peer-reviewed journals—particularly in recent years. This growth is consistent with the action from the HSE patient safety strategy “to support patient safety research and publish and act on the results” (p.19]) [4]. The majority of the research on MMS in the RoI was focused on measuring past harm (particularly adverse events), and anticipation and preparedness (particularly assessments of safety culture/climate). Most of the intervention studies were concerned with education and training. We will make recommendations for areas of future research based on the findings from the scoping review, and identify how these recommendations align with relevant aims from the HSE patient safety strategy 2019–2024 [4].

The focus on adverse events as a method of measuring past harm is consistent with the substantial increase in research publications on this approach to measuring safety in healthcare [49]. Staff surveys are a commonly used source of information on adverse events. However, a survey approach is constrained by the extent to which conclusions can be drawn about adverse event prevalence. Patient record review has been considered the “gold standard” patient safety research method [50], and was used in four of the reviewed studies. Such data are useful in demonstrating the scope of the problem in the Irish healthcare system, allows for international comparisons, and for an assessment of any changes over time. However, patient record review data are limited in terms of identifying specific areas for safety improvement [50, 51]. Therefore, there is a need for measures tailored to distinct aspects of patient harm (e.g. specific care-related injuries, missed diagnoses that lead to harm) [50]. Such data is important to address the HSE goal to “measure and monitor safety, to evaluate the effects of safety improvement initiatives, and to inform further emerging priorities”(p.19) [4]. Data on specific aspects of patient harm will allow the alignment of adverse events with failures in care, and the development and evaluation of interventions to address these issues [52].

Safety culture/climate surveys were the most frequently used approach to measuring and monitoring anticipation and preparedness. Again, this is consistent with the large amount of research devoted to these types of measures more broadly in the safety literature [53, 54]. Safety culture/climate data is useful in identifying areas of both strength and weakness. However, it has been suggested that such survey measures may be best viewed as a trusted “wet finger” to find out which way the wind blows [55], and do not identify specific areas for improvement. To illustrate, working conditions were identified as an area for improvement across four of the included studies [34–37]. However, further data is required to identify the specific working conditions that should be prioritised for change. This is why, in some safety culture interventions, the survey data is used to inform discussion in qualitative safety culture workshops to identify the specific issues that need to be addressed [56]. It is recommended that future research should consider how to measure safety culture/climate in a way that is practical, sufficiently specific to identify areas for safety improvement, and can be used to measure whether improvements have occurred. This will likely require a combination of quantitative and qualitative data collection methodologies. A consideration of how to measure safety culture/climate is particularly important in the RoI as this has been identified as a specific action in the HSE patient safety strategy [4].

Compared to the MMS dimensions of past harm and anticipation and preparedness, a lower number of studies in our scoping review were concerned with MMS in the other three safety dimensions—particularly integration and learning. These proportions are similar to the findings from a systematic review of MMS in prehospital care [51]. Although the studies in our scoping review that assessed one of these three dimensions of MMS provided informative data, they were largely based upon staff survey responses. Only one study [41] utilised clinical data. It is suggested that consideration should be given to the identification of feasible methods to MMS in these three under-researched dimensions beyond that derived only from survey data. A robust safety surveillance system should comprise multiple methods and address all five MMS domains. Research is recommended to critically appraise the existing safety monitoring system in the RoI healthcare system in order to identify blind spots as well as where there may be duplication of effort. Such research is consistent with the HSE patient safety strategy aim to “further develop and enhance local and national suites of key patient safety indicators” (p. 19) [4].

Although MMS is important, what is also essential is that this data is used to identify and evaluate the effectiveness of interventions to improve patient safety and quality of care [52]. In fact, there is arguably little point in collecting safety data if it is not then used to bring about improvement. Three out of the six safety interventions identified were focused on education and training—a person-focused intervention at the lowest level of the hierarchy of intervention effectiveness [15]. Although two interventions [45, 46] with a focus on simplification and standardisation were identified, no interventions were found at the highest two levels of the hierarchy—forcing functions, automation and computerisation. The evaluations of the interventions included in the review were positive. However, similar to the majority of assessments of patient safety interventions, the quality of the evidence of effectiveness was low, with limited evidence of an impact on patient outcomes [5, 57, 58]. It is recommended that future research focuses on the evaluation of more effective system-focused interventions. It is further recommended that interventions are closely aligned to appropriate, and meaningful, measures of MMS in order to support rigour in evaluation of the impact of interventions on patient safety. This alignment will be necessary to achieve the HSE patient safety aims of putting in place appropriate actions to mitigate risks to patients, prioritising specific safety improvement initiatives, and evaluating the effects of safety improvement and risk mitigation initiatives [4]. It is also suggested that the co-design approach used by Ward et al. [48] may offer a useful approach to identify specific interventions that healthcare staff believe will improve safety.

## Limitations

There are a number of limitations to our scoping review. Firstly, a quality assessment was not carried out, although this absence is consistent with the majority of other scoping reviews [59]. Secondly, our scoping review provided a more descriptive summary of the literature than would be the case from a systematic review. This is a result of the goal of a scoping review to provide a map of existing research, rather than to answer a specific question [60]. Thirdly, as in any synthesis of the literature, scoping reviews are at risk for bias [60]. Fourthly, studies that focused on patient safety in the context of patients with a particular medical condition or focused on the safety of one process were excluded from our review. The rationale for this exclusion was that it would have been impossible to devise a search strategy that included every possible medical condition, and process. Therefore, we chose to take an approach that included all papers that met the inclusion criteria rather than an approach that, although broader, may have missed particular studies. Finally, we did not carry out a search of the grey literature. These searches were not carried out as there are methodological issues with including grey literature searches in systematic reviews (e.g. compromised methodological reproducibility, difficulties in interpreting these publications [61]).

## Conclusion

There is a modest, but growing, body of patient safety research conducted in the RoI. This scoping review has demonstrated the variety of patient safety research being carried out in the RoI. It is hoped that this review will provide direction to researchers, healthcare practitioners, and health service managers, in how to build upon the existing research in order to improve patient safety and quality of care.
